# Solvent Polarity Engineering in Low-DMF ZIF-7 Membrane Growth: Crystallization Behavior, Heterogeneous Intergrowth, and Microstructural Evolution

**DOI:** 10.3390/molecules31132348

**Published:** 2026-07-03

**Authors:** Fernando Romero-Romero, Sergio Armando Serrano-Palafox, Vidal Morales-Mercado, Murali Venkata Basavanag Unnamatla, José Miguel Arriaga-Merced, Maria Fernanda Ballesteros-Rivas, Victor Varela-Guerrero

**Affiliations:** 1 Facultad de Química, Universidad Autónoma del Estado de México, Paseo Colón y Paseo Tollocan S/N, Toluca 50120, Estado de Mexico, Mexico; mvbasavanagu@uaemex.mx (M.V.B.U.); mfballesterosr@uaemex.mx (M.F.B.-R.); 2 Centro Conjunto de Investigación en Química Sustentable UAEM-UNAM, Carretera Toluca-Atlacomulco, km 14.5, Toluca 50200, Estado de Mexico, Mexico; sserranop003@alumno.uaemex.mx (S.A.S.-P.); vmoralesm@uaemex.mx (V.M.-M.); jmiguel_arriagam@yahoo.com.mx (J.M.A.-M.)

**Keywords:** ZIF-7 membranes, solvent polarity engineering, microstructural evolution, heterogeneous crystallization

## Abstract

Molecular transport membranes are promising alternatives to conventional cryogenic separation processes. Here, solvent polarity effects were investigated by varying the DMF/MeOH ratio during the solvothermal synthesis of supported ZIF-7 membranes. A DMF:MeOH ratio of 1:3 preserved the characteristic sodalite topology while suppressing dense-phase formation. Methanol incorporation modified heterogeneous crystallization behavior, intercrystalline organization, membrane morphology, and film densification on α-alumina supports while reducing DMF consumption by approximately 75%. These effects are associated with solvent-mediated precursor solvation, Zn^2+^–benzimidazole coordination equilibria, and heterogeneous nucleation at the support–solution interface. Although low BET surface areas were obtained from N_2_ adsorption at 77 K, these values were interpreted cautiously considering the known limitations of nitrogen physisorption in flexible ultramicroporous frameworks. Overall, the results support solvent polarity engineering as a physicochemical strategy for regulating membrane microstructural evolution under reduced DMF conditions. Accordingly, the transport behavior discussed herein is interpreted primarily from a solvent-mediated microstructural perspective rather than as a direct quantitative descriptor of accessible porosity.

## 1. Introduction

Membrane technology has become an attractive alternative for molecular transport separation. Energy consumption in membrane separation process is lower than in typical adsorption and cryogenic distillation methods [1,2]. In addition, membranes have an outstanding stability under a wide range of operating conditions, as well as both effortless control and scale-up for their application in H_2_, N_2_, O_2_, CO_2_ and CH_4_ separation at industrial level [3,4,5,6]. However, polymeric and inorganic membranes possess both fragile and thermal unstable structures, which results in poor selectivity [7,8].

Metal–organic framework (MOF)-based membranes, particularly zeolitic imidazolate framework (ZIF), have emerged as an alternative for selective molecular transport applications due to their structural properties, as well as both their thermal and chemical stability [9,10]. MOFs allow tuning their properties with the right combination of metallic centers and organic ligands, resulting in a broad range of applications such as storage and gas separation [11,12,13,14,15,16,17,18,19,20,21,22,23], sensors [24,25] and heterogeneous catalysis [26,27,28].

Huang et al. [29] reported the first synthesis of the ZIF-7 structure. The zinc atoms were bridged to a benzimidazole ligand bringing a sodalite topology. ZIF-7 membranes have attracted considerable attention for gas separation due to their size-dependent transport behavior and diffusivity-based transport differentiation mechanisms [30,31].

ZIF-7 membranes, grounded on α-alumina, are commonly synthesized via in situ growth [32,33], secondary growth [34] or electrospray [35]. N,N-dimethylformamide (DMF) is a typical solvent used for ZIF-7 syntheses under solvothermal conditions. However, long activation times and controlled temperatures are required to avoid membrane’s fracture when guest solvent molecules are extracted from cavities. In addition, it must be highlighted that DMF is an expensive organic solvent, with high potential for both environmental pollution and human health affection. DMF has been identified as a high-risk solvent according to green chemistry metrics, encouraging the search for safer and more sustainable alternatives [36,37,38].

Recent studies have explored DMF-free synthesis routes using water–methanol systems. Even though the obtained structures belong to a large pore ZIF-7 phase, a transition to small pore phase is noted at temperatures higher than 50 °C [30]. If only water is used as a solvent, more benzimidazole ligand is needed in ZIF-7 synthesis [39]. Tu et al. [40] reported that a DMF/MeOH solvent ratio of 1:1 works for the ZIF-7 synthesis. Nevertheless, no higher dilution ratios have been considered in ZIF-7 syntheses, in order to lessen the amount of DMF. To the best of our knowledge, DMF-free or low-DMF ZIF-7 membrane fabrication remains scarcely reported. This work bridges the gap between solvent composition and physicochemical control of membrane formation, providing a rational strategy for tuning membrane microstructure and transport behavior through solvent polarity engineering. Although water-based systems have been explored, they often lead to phase transitions or require excess ligand concentrations, limiting their applicability for controlled ZIF-7 membrane synthesis.

Despite these advances, most studies still rely on DMF-dominated systems, with limited exploration of solvent polarity tuning as a strategy to control ZIF-7 morphology and transport behavior [29,33]. Given the environmental and safety concerns associated with DMF, the development of alternative solvent strategies is highly desirable. In this work, we propose a solvent polarity modulation approach by partially replacing DMF with methanol to control nucleation and crystal growth during ZIF-7 membrane formation. Therefore, solvent molecules play an active role in determining the coordination environment and assembly pathway, ultimately governing membrane microstructure and gas transport behavior [41,42]. Unlike previous studies that primarily focus on solvent composition, this work investigates how DMF/MeOH solvent polarity engineering influences ZIF-7 crystallization behavior, heterogeneous membrane growth, morphology evolution, film thickness, and selective transport behavior. Rather than relying exclusively on textural porosity as the central descriptor, this study emphasizes solvent-mediated crystallization control as a strategy to reduce DMF consumption while preserving comparable heterogeneous growth and membrane densification tendencies associated with ZIF-7 membrane growth. In the present system, the DMF/MeOH mixed solvent is not treated merely as a compositional variable but as a physicochemical regulator of precursor speciation and crystallization kinetics. DMF acts as a high-boiling polar aprotic solvent that can coordinate to Zn^2+^ centers through the oxygen atom of its carbonyl group, behaving as an ancillary ligand and influencing the coordination environment during framework assembly, as reported for coordination polymers and MOF systems [43]. In contrast, methanol, as a polar protic solvent, can also interact with Zn^2+^ through its oxygen atom, while simultaneously modifying hydrogen-bonding interactions and the solvation environment of benzimidazole, thereby affecting ligand availability and coordination equilibria [40,44]. Benzimidazole, as an N-donor ligand, strongly coordinates to Zn^2+^ through its nitrogen atoms, forming stable metal–ligand bonds that define the framework structure [45]. In this context, the mixed solvent system not only controls the apparent polarity of the medium but also participates in coordination and solvation phenomena, influencing precursor dispersion, ligand exchange dynamics, nucleation rate, and crystal growth behavior.

In MOF and ZIF synthesis, solvent composition should not be considered only as a reaction medium, but also as a physicochemical variable capable of modifying precursor solvation, metal–ligand exchange, supersaturation, nucleation density, and crystal growth pathways. In supported membrane systems, these solvent-mediated effects become even more relevant because crystallization occurs under heterogeneous conditions at the support–solution interface. Therefore, solvent polarity engineering provides a rational route to control not only crystal formation, but also membrane continuity, intercrystalline packing, and transport pathways [41,46].

In this context, the polarity of the solvent system is modulated by varying the DMF:MeOH ratio, considering that DMF is a polar aprotic solvent (dielectric constant ≈ 36.7) and methanol is a polar protic solvent (dielectric constant ≈ 32.6). The progressive increase in methanol content alters solvation interactions and coordination equilibria with the benzimidazole linker, thereby influencing nucleation and crystal growth processes. To further address the role of solvent polarity in the mixed system, the effective dielectric constant ($\varepsilon_{mix}$) was estimated using a linear mixing approximation $\left(\varepsilon_{mix}=x_{DMF}\cdot \varepsilon_{DMF}+x_{MeOH}\cdot \varepsilon_{MeOH}\right)$. For the DMF:MeOH ratio of 1:3, $\varepsilon_{mix}$ ≈ 33.7, which lies between those of pure DMF and methanol. This intermediate polarity provides a quantitative basis for understanding how the mixed solvent system modifies solvation interactions, coordination equilibria, and nucleation kinetics, directly impacting crystal growth and membrane microstructure. Although the effective dielectric constant provides a first quantitative approximation, solvent effects in ZIF crystallization are not governed solely by polarity. Hydrogen-bond donation ability, solvent–metal coordination, and precursor solvation also play a decisive role [40,44]. In this context, methanol modifies the hydrogen-bonding environment of benzimidazole, while DMF maintains solvation and dispersion of the zinc precursor, resulting in a combined solvent-mediated control of nucleation and growth processes [41,42]. Accordingly, the present work was designed to investigate the influence of solvent polarity on supported ZIF-7 membrane growth and heterogeneous intergrowth, rather than to establish a comprehensive porosity characterization study.

## 2. Results and Discussion

### 2.1. Synthesis of ZIF-7

**Figure 1:**
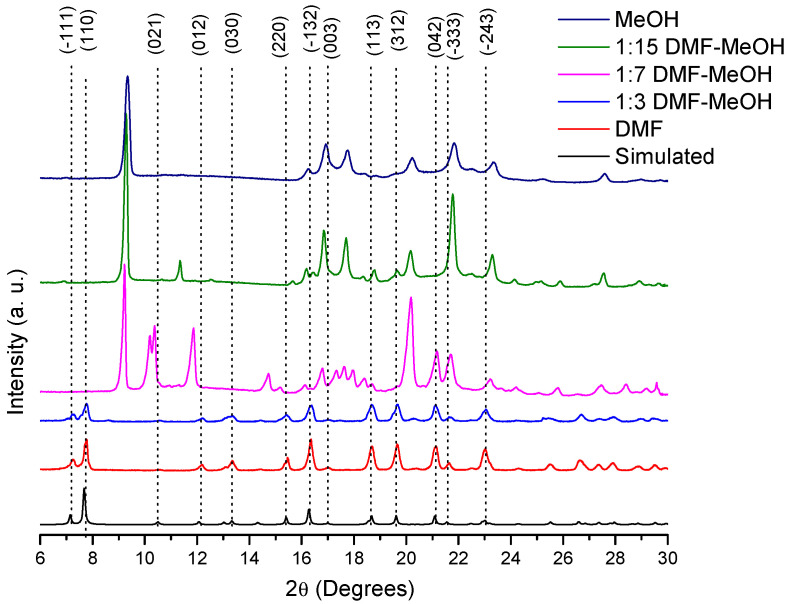
XRD patterns of ZIF-7 synthesized at different DMF:MeOH ratios (synthesis time: 3 h).

Figure 1 shows the X-ray diffraction patterns of the synthesized ZIF-7 at different DMF:MeOH ratios. It can be observed that when only MeOH and DMF:MeOH ratios are above 1:3, the porous ZIF-7 phase is not formed. A dense phase is formed instead (as reported by Yang et al.) [47]. On the other hand, when only DMF and a DMF:MeOH solvent ratio of 1:3 was used in the synthesis process, the characteristic peaks of the ZIF-7 phase are well defined at 2θ = 7.14, 7.68, and 10.42°. The Zn–N vibration band at approximately 422 cm^−1^ was assigned and labeled in Figure S2 for the samples in which the ZIF-7 crystalline phase was obtained. For the pure methanol system, crystalline ZIF-7 was not formed under the present synthesis conditions; therefore, a representative FTIR spectrum of crystalline ZIF-7 synthesized in pure methanol could not be provided. The observed peak broadening and slight shift in the XRD pattern of the mixed solvent samples indicate partial structural disorder and reduced crystal size, consistent with previously reported correlations between diffraction peak distortion and framework disorder in metal–organic structures [48].

**Figure 2:**
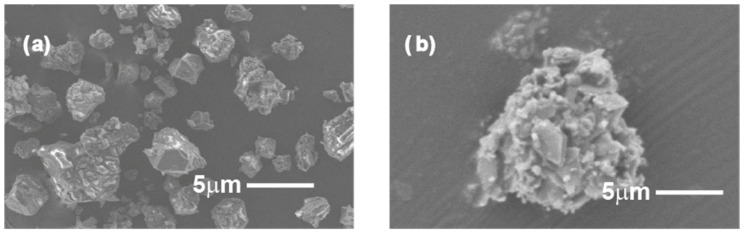
Micrographs of ZIF-7. (**a**): DMF. (**b**) DMF:MeOH (1:3).

Figure 2 shows the morphology of the ZIF-7 crystals for both the DMF solvent (ZIF-7-D) and the mixture of solvents at 1:3 ratio (ZIF-7-M). Crystal sizes of about 2 μm are obtained when only DMF is used (Figure 2a). In the mixed DMF:MeOH system, smaller and more irregular crystal aggregates were observed within the analyzed SEM region. Since the observed morphology shows local heterogeneity, the description was revised to avoid overgeneralization of crystal size and distribution across the entire sample (Figure 2b). The reduction in crystal size observed in the mixed DMF:MeOH system may arise from solvent-mediated changes in precursor solvation, ligand availability, and coordination equilibria. Methanol, as a polar protic solvent, can modify hydrogen-bond interactions with benzimidazole and alter the local solvation environment around Zn^2+^ species. This may delay supersaturation, reduce crystal growth rates, and promote the formation of smaller nuclei. Therefore, the mixed-solvent system should be interpreted not simply as a dilution strategy, but as a crystallization-control medium that regulates nucleation density, crystal growth, and intercrystalline organization [48,49].

According to TGA results, the ZIF-7-D structure presents a weight loss of 13% registered at 150 °C related to the evaporation of the DMF solvent molecules. At temperatures above 450 °C the thermal stability of such structure becomes compromised (Figure S3a). On the other hand, the ZIF-7-M registered a weight loss of 12% between 30 °C and 100 °C related to MeOH evaporation. Between 130 °C and 200 °C, a weight loss of 21%, attributed to DMF evaporation takes place. A complete degradation of the ZIF-7-M structure takes place at temperatures above 500 °C (Figure S3b). Compared to membranes synthesized in pure DMF systems reported in the literature, which typically exhibit stability ranges between 450–480 °C, the 1:3 DMF:MeOH membrane demonstrated comparable or slightly enhanced thermal resistance, indicating that partial solvent substitution does not compromise structural robustness. Although DTG curves would provide a more quantitative distinction between solvent removal, ligand decomposition, and framework collapse, the TGA profiles provide qualitative evidence of the main mass-loss regions associated with residual solvent removal and framework degradation. Therefore, the thermal analysis is discussed qualitatively, and this limitation is explicitly acknowledged.

The BET surface areas obtained from N_2_ adsorption at 77 K were 2.3 and 2.1 m^2^/g for ZIF-7-D and ZIF-7-M, respectively (Table 1). These values are significantly lower than those commonly reported for activated ZIF-7 powders and therefore must be interpreted cautiously. ZIF-7 is a flexible ultramicroporous framework, and N_2_ adsorption at 77 K may be limited by restricted diffusion, narrow pore apertures, and gate-opening effects. Consequently, BET surface area is not used here as the primary descriptor of membrane functionality. Instead, the interpretation focuses on the relationship between solvent composition, crystallization behavior, membrane morphology, film thickness, intercrystalline packing, and microstructural diffusion behavior [50,51,52]. Therefore, the low N_2_-derived BET values are treated as a limitation of textural characterization rather than as the central criterion for evaluating the solvent-mediated membrane growth strategy. The authors acknowledge that complementary CO_2_ adsorption measurements at 273 K would provide a more complete evaluation of ultramicroporous accessibility in ZIF-7. Likewise, DTG curves and complete N_2_ adsorption–desorption isotherms with pore size distribution analyses would further strengthen the quantitative comparison of solvent removal, framework decomposition, and pore accessibility. Nevertheless, the present work was intentionally focused on understanding how solvent polarity engineering modulates heterogeneous crystallization behavior, membrane growth kinetics, intercrystalline organization, morphology evolution, membrane densification, and transport pathway regulation under reduced DMF conditions. Accordingly, the conclusions were carefully revised to avoid overinterpretation of BET surface area data and to emphasize solvent-mediated membrane formation and heterogeneous intergrowth rather than absolute porosity determination. These considerations are particularly relevant for flexible ultramicroporous ZIF systems, where adsorbate accessibility and transport behavior may depend not only on intrinsic pore dimensions but also on framework flexibility and mesoscale membrane organization. Future studies involving advanced adsorption characterization and in situ crystallization analysis could provide further insight into ultramicropore accessibility and solvent-mediated growth dynamics in supported ZIF-7 membranes. Therefore, the present work should not be interpreted as a definitive adsorption-based porosity study, but rather as a mechanistic analysis of solvent-mediated membrane crystallization and heterogeneous intergrowth.

### 2.2. ZIF-7 Membrane by In Situ Growth

**Figure 3:**
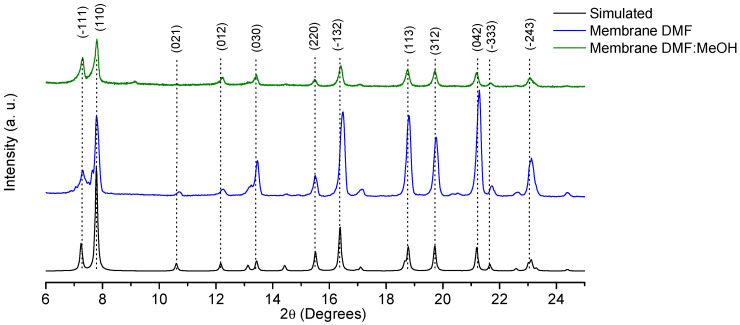
XRD pattern of ZIF-7 membranes. DMF:MeOH and DMF only.

Following the in situ growth procedure, ZIF-7 membranes grounded on α-alumina were synthesized with both DMF (Membrane 1) and MeOH: DMF with a ratio of 1:3 (Membrane 2). The X-ray diffraction patterns are shown in Figure 3. Pure DMF results in sharper reflections, indicating higher crystallinity. So, it can be stated that the degree of crystallinity of the ZIF-7 structures is highly related to the use of aprotic solvents. Minor additional peaks observed in the DMF-derived membrane may be attributed to partial structural disorder or residual phases, as commonly reported in solvothermal MOF synthesis.

**Figure 4:**
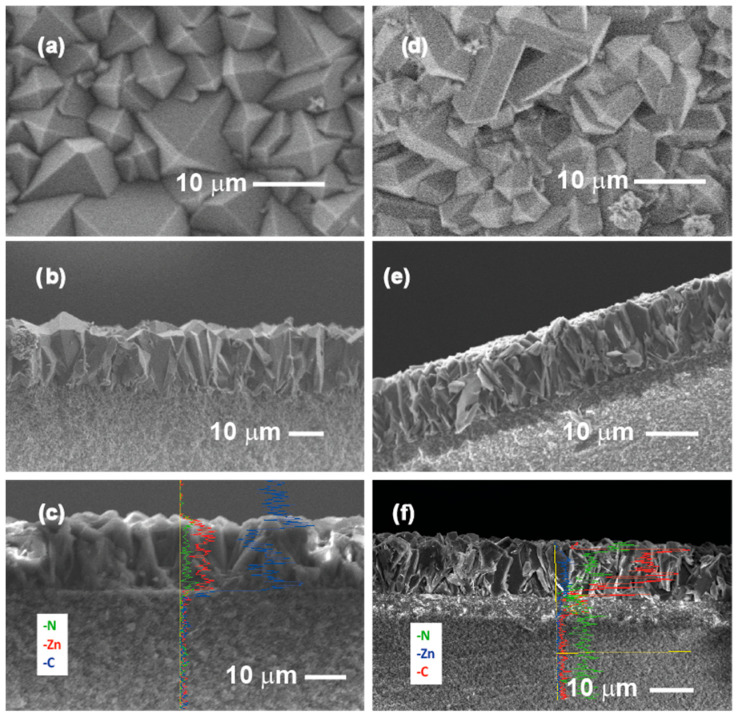
SEM micrographs of ZIF-7 membrane. (**a**) DMF (**d**) DMF:MeOH: Surface. (**b**,**e**): Cross-section. (**c**,**f**): Element profile.

Significant differences in surface morphology between ZIF-7 membranes synthesized in DMF (Membrane 1) and DMF:MeOH (Membrane 2) were noticed during the electron microscopy analysis, particularly in terms of crystal size and membrane–support interaction (Figure 4). A top view of Membrane 1 shows a fully covered surface with crystals of approximately 15 μm, while its cross-section reveals a uniform thickness of around 15 μm (Figure 4a,b). In contrast, Membrane 2 exhibits a more random distribution of ZIF-7 crystals across the substrate surface, with crystal sizes below 12 μm and an average thickness of approximately 11 μm. The prior modification of the α-alumina support with the organic linker promotes heterogeneous nucleation by increasing the density of anchoring sites on the substrate surface [31]; However, the observed reduction in crystal size and thickness in Membrane 2 suggests that the solvent composition plays a decisive role in modulating nucleation and growth kinetics. In particular, the DMF/MeOH ratio appears to influence crystal growth behavior, likely through polarity effects that affect coordination equilibria and crystal growth rates, rather than acting strictly as a classical structure-directing agent [44].

The difference in crystal sizes observed in Figure 4a,d is therefore attributed to solvent effects during membrane synthesis. In this sense, when the polar aprotic solvent is only used (DMF), crystal growth may proceed more rapidly. In contrast, the presence of a polar protic solvent (methanol) can modify nucleation kinetics through solvation effects on the benzimidazole linker [53]. The establishment of hydrogen bonds between methanol and benzimidazole leads to the creation of a barrier around the binder, restricting its interaction with the deprotonating agent and, as a result, its coordination with Zn^2+^ ions [54], potentially leading to slower crystal growth. As a consequence, reduced growth rates may favor the formation of smaller crystals [55]. The occurrence of smaller crystals in Membrane 2 is associated with the development of thinner films (Figure 4b,e) and a more compact intercrystalline packing with reduced void spaces between adjacent crystals. To further address reproducibility, membrane thickness measurements were performed on at least three independently synthesized samples. The average thickness of Membrane 1 was 15 ± 1.5 µm, while Membrane 2 exhibited an average thickness of 11 ± 1.2 µm. These results confirm that the observed differences in membrane thickness are consistent and reproducible, and directly related to solvent composition rather than experimental variability. The relationship between membrane thickness and gas selective pathway organizations should not be interpreted solely in terms of diffusion resistance. Solvent-mediated growth may simultaneously influence membrane densification, intercrystalline connectivity, crystal intergrowth, and non-selective transport pathways. Therefore, the lower permeance and more differentiated H_2_/CO_2_ microstructure-associated transport behavior observed for Membrane 2 may reflect not only thickness differences, but also a denser microstructure and reduced defect-mediated transport.

Membrane formation on α-alumina should be interpreted as a heterogeneous crystallization process rather than a direct replication of powder crystallization. The modified support provides anchoring sites for benzimidazole-containing species, while the macroporous structure of α-alumina may allow partial precursor penetration. Under these conditions, local supersaturation, ligand availability, and the Zn^2+^ coordination environment near the support surface may differ from those in bulk solution. Consequently, membrane continuity, crystal intergrowth, and intercrystalline transport pathways are strongly influenced by the support–solution interface [56].

The differences between the morphologies observed in Figure 2 and Figure 4 arise from the different crystallization environments. Figure 2 corresponds to ZIF-7 crystals formed in bulk solution, where nucleation occurs without the influence of a solid substrate. In contrast, Figure 4 corresponds to ZIF-7 membrane growth on modified α-alumina, where heterogeneous nucleation, surface anchoring, precursor penetration into macropores, and substrate–solution interactions influence crystal orientation, packing, apparent size, and film thickness. Therefore, powder morphology and supported membrane morphology should not be interpreted as directly equivalent.

**Figure 5:**
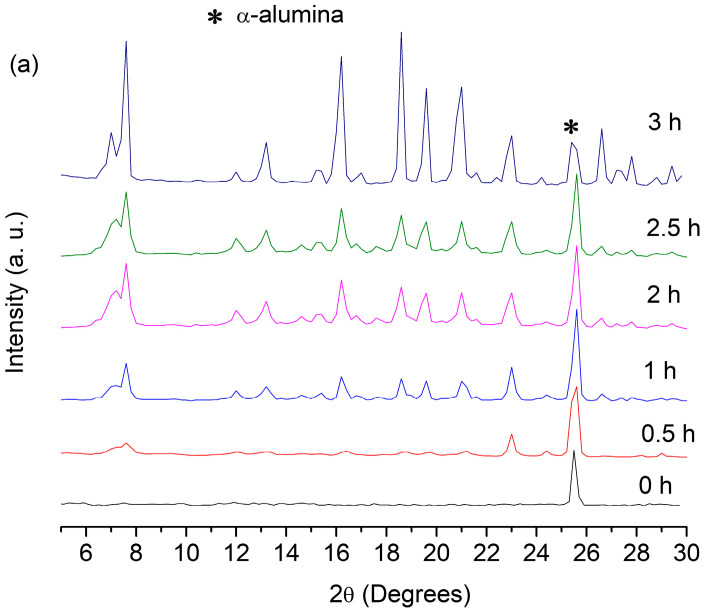

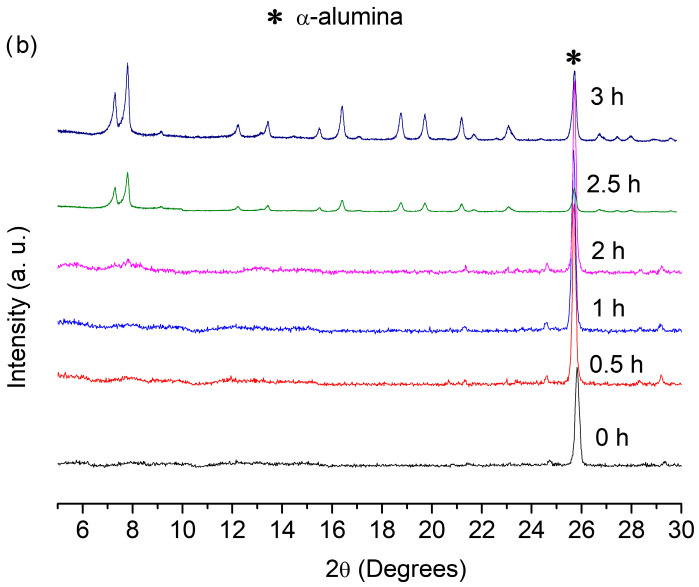
XRD patterns of ZIF-7 membranes at different reaction time. (**a**): Membrane 1 (DMF). (**b**): Membrane 2 (DMF:MeOH).

A series of membranes using DMF and a DMF:MeOH ratio of 1:3 were synthesized at 0.5, 1, 2 and 2.5 h of reaction time in order to evaluate the growth of the ZIF-7 crystals on the α-alumina support. Figure 5 shows the X-ray diffraction patterns obtained for both systems. A fast growing of ZIF-7 crystals is noticed after the first hour when only DMF is used as a solvent (Figure 5a). With the solvent mixture, the growing of ZIF-7 crystal begins after two hours.

**Figure 6:**
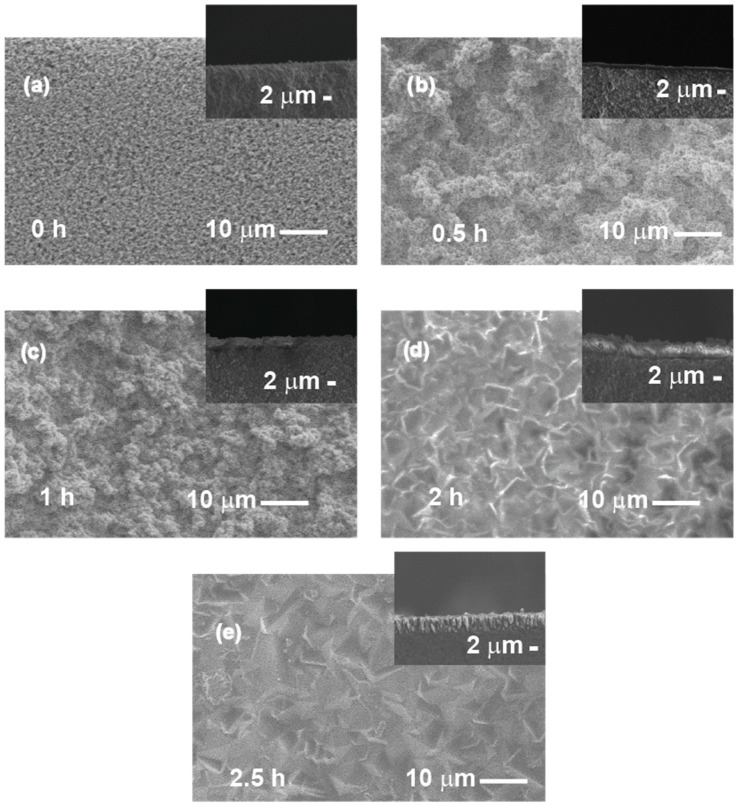
Electron micrographs of the top view and cross-section of ZIF-7 membranes (DMF) synthesized at different time. (**a**): 0 h, (**b**): 0.5 h, (**c**): 1.0 h, (**d**): 2 h, (**e**) 2.5 h.

**Figure 7:**
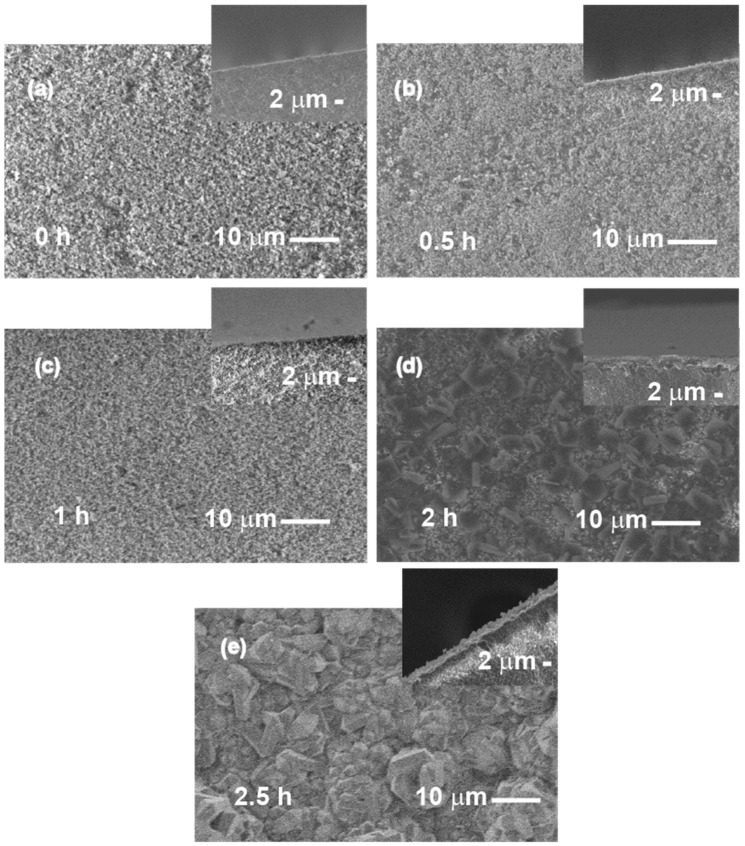
Electron micrographs of the top view and cross-section of ZIF-7 membranes (DMF:MeOH, 1:3) synthesized at different time. (**a**): 0 h, (**b**): 0.5 h, (**c**): 1.0 h, (**d**): 2 h, (**e**) 2.5 h.

The top and cross-sectional views of DMF and DMF:MeOH membranes at different time are shown in Figure 6 and Figure 7. A significant change in surface morphology is noticed after 0.5 and 2 h for DMF and DMF:MeOH membranes, respectively, which correlates with the results obtained by X-ray diffraction.

The aforementioned findings evidence the solvation effect caused by the presence of a polar protic solvent such as methanol, which limits the interaction between benzimidazole and sodium formate, resulting in a slow crystal growth rate. The slower crystallization observed in the mixed-solvent system is consistent with nucleation and growth trends commonly associated with LaMer-type behavior. However, because direct in situ kinetic measurements were not performed, this interpretation is presented as a plausible mechanistic framework rather than definitive experimental proof of the LaMer model, since the protic component retards supersaturation build-up and delays the onset of the nucleation [41]. In particular, the DMF/MeOH ratio appears to influence crystal growth behavior through solvent-mediated effects on coordination equilibria and nucleation kinetics, rather than acting as a classical structure-directing agent [40]. Therefore, the mixed solvent influences crystal growth by modulating nucleation kinetics and coordination equilibria, controlling membrane thickness and microstructure [42]. This interpretation is consistent with the observed reduction in crystal size and membrane thickness in the mixed solvent system, indicating a slower and more controlled nucleation–growth process compared to the pure DMF system.

### 2.3. Evaluation of Permeability

Membranes were activated by the solvent exchange method. Permeation tests were performed to evaluate selectivity in a single component system (H_2_, CO_2_, N_2_, C_3_H_8_, and C_3_H_6_). Experiments were carried out at room temperature and high vacuum using the conventional *time-lag* method at 2.02 × 10^5^ Pa. Permeation values and qualitative H_2_/CO_2_ transport differentiation behavior obtained with Membrane 1 and Membrane 2 are presented in Table 2. Variations observed in permeance values (Table 2) are attributed to experimental variability between independently prepared membranes, confirming the importance of reproducibility in membrane synthesis. Membrane 2 exhibited more restricted transport behavior than Membrane 1, particularly for CO_2_, N_2_, and C_3_H_8_. The gas permeation results are interpreted qualitatively in terms of diffusion behavior, rather than definitive proof of fully accessible microporosity. Membrane 2 showed lower permeance and more differentiated microstructure-associated diffusion behavior than Membrane 1. This behavior is plausibly associated with the combined influence of film thickness, intercrystalline packing, reduced non-selective pathways, and solvent-mediated membrane growth. Therefore, the more differentiated H_2_/CO_2_ transport pathway organization is interpreted primarily as a consequence of solvent-mediated membrane microstructural evolution, intercrystalline packing, and transport pathway regulation, rather than being attributed exclusively to intrinsic textural porosity [31,57]. Although H_2_ has a smaller kinetic diameter (2.89 Å), CO_2_ exhibits stronger adsorption interactions with the ZIF-7 framework due to its higher quadrupole moment and polarizability. In addition, the framework characteristics and intercrystalline transport restrictions may contribute to size-dependent transport behavior, potentially favoring differentiated transport behavior. The modified intercrystalline packing in Membrane 2 may contribute to more restricted non-selective transport pathways, thereby favoring the observed transport differentiation trends.

**Figure 8:**
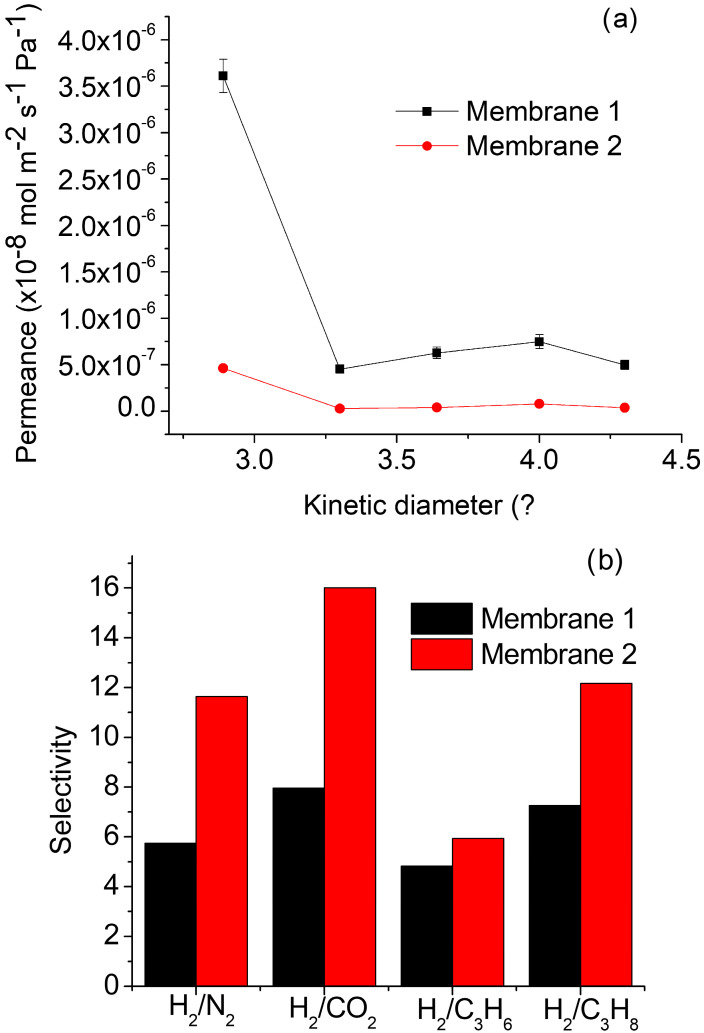
(**a**) Permeance and qualitative transport differentiation trends as a function of kinetic diameter with ZIF-7 membrane. Membrane 1: DMF. Membrane 2: DMF: MeOH (**b**) Selectivity.

In addition to the differentiated transport behavior, Membrane 2 also showed a differentiated diffusion behavior toward the gases evaluated (Figure 8a,b). The heterogeneous growth observed in Membrane 2 can be rationalized by considering the combined effects of support functionalization and solvent polarity. While the organic linker modification increases the number of nucleation sites, the presence of methanol modifies coordination equilibria and slows crystal growth kinetics. This combination may promote distributed nucleation events with variable growth rates across the substrate surface, resulting in heterogeneous crystal development (Figure 4), potentially contributing to a more compact membrane microstructure with more restricted non-selective transport pathways. It is plausible that due to the longer time needed for crystal growth in Membrane 2, partial crystal growth within the support structure may occur. This behavior may plausibly contribute to partial crystal growth within the support structure, resulting in a more compact transport region that may restrict non-selective transport of larger molecules.

The qualitative H_2_/CO_2_ transport differentiation trends observed for Membrane 2 show qualitative agreement with transport evolution previously reported by Go et al. [57] and Noh et al. [33], which are 15.98 and 15.2, respectively, for membranes prepared via the in situ growth method. Notably, the qualitative transport differentiation trends observed in this study support the role of solvent composition in regulating membrane microstructure and transport pathway organization. A key advantage of the proposed membrane synthesis strategy is the substantial reduction in DMF usage through partial substitution with methanol, in contrast to conventional DMF-dominated protocols. Importantly, these solvent-mediated effects were achieved while maintaining comparable membrane densification tendencies and thermal stability under the evaluated conditions. These findings support the role of solvent polarity modulation in regulating nucleation kinetics, membrane microstructure, and transport pathway organization.

The difference between Membrane 1 and Membrane 2 should be interpreted as the result of solvent-mediated mesoscale organization. In polycrystalline MOF membranes, gas transport is not governed exclusively by intrinsic pore dimensions, but also by intercrystalline boundaries, defect density, film thickness, and support–membrane integration. In the present system, the mixed DMF:MeOH solvent likely modifies nucleation and growth rates, leading to changes in crystal packing and intergrowth. These microstructural changes may contribute to more restricted non-selective transport pathways, explaining the lower permeance and more differentiated H_2_/CO_2_ transport tendencies observed for Membrane 2.

Additionally, transport in polycrystalline MOF membranes is strongly influenced by grain boundaries, intercrystalline defects, crystal intergrowth, and membrane densification. Therefore, transport behavior may reflect the combined contribution of intrinsic framework characteristics and mesoscale microstructural organization rather than exclusively the accessible microporosity determined from N_2_ adsorption measurements. This behavior is particularly relevant in polycrystalline MOF membranes, where transport may arise from the coupled contribution of framework accessibility, intercrystalline packing, grain boundary organization, and defect-mediated pathways. Therefore, gas diffusion trends should be interpreted as a consequence of the overall membrane microstructure rather than as a direct function of BET surface area alone.

As shown in Table 3, the apparent transport differentiation trends observed in this study show qualitative agreement with diffusion behavior previously reported for ZIF-7 membranes synthesized using conventional DMF-based systems. Although comparisons with literature should be interpreted cautiously due to differences in synthesis conditions and membrane configurations, the present results suggest that qualitatively comparable diffusion behavior was achieved with significantly reduced DMF consumption. These findings support solvent polarity modulation as a physicochemical strategy for regulating membrane densification and transport pathway organization.

## 3. Materials and Methods

### 3.1. Materials

Zinc nitrate hexahydrate (Zn(NO_3_)_2_·6H_2_O, >98% Sigma-Aldrich, Toluca, Mexico), benzimidazole (bim, C_7_H_6_N_2_, >98% Sigma-Aldrich), and sodium formate (NaCOOH) were employed as the metal source, the ligand, and the deprotonation agent, respectively. N, N-dimethylformamide (DMF, HCON(CH_3_)_2_, >99% Sigma-Aldrich), and methanol (MeOH, CH_3_OH, >98% J. T. Baker, Guadalajara, Mexico) were utilized as solvents. All chemicals were utilized as received without further purification.

### 3.2. Synthesis of ZIF-7

The synthesis route proposed by McCarthy et al. [32], was used to grow ZIF-7 crystals. A binary DMF/MeOH solvent system was prepared at volume ratios of 1:0, 1:3, 1:7, 1:15, and 0:1. A solid mixture 5.14 mmol of Zn(NO_3_)_2_·6H_2_O, 6.86 mmol of bim and 15.21 mmol of NaCOOH, was dissolved in 40 mL of the DMF/MeOH solvent mixture and kept under stirring for 20 min at room temperature. After that, the solution was placed in an autoclave and heated at 120 °C for 3 h. The 120 °C solvothermal temperature was selected to ensure complete ligand coordination while avoiding ZnO formation, as reported in [32]. The resulting precipitate was rinsed and centrifuged three times with methanol and allowed to dry overnight at 60 °C.

Methanol was selected as the washing solvent due to its lower boiling point and high volatility, which facilitate efficient removal of residual DMF molecules from the framework cavities through solvent exchange. Although thermal activation can also remove guest molecules, rapid solvent evaporation at elevated temperatures may induce structural stress and lead to crack formation in supported membranes. Therefore, solvent exchange using methanol provides a milder and more controlled activation route, preserving membrane integrity and avoiding defect formation. This approach is widely reported as an effective activation method for ZIF materials.

### 3.3. Synthesis of ZIF-7 Membrane by In Situ Growth

#### 3.3.1. Modified α-Alumina Support

An α-alumina buttress of 22 mm diameter and 2 mm thickness was previously dried at 200 °C for 2 h. Thereafter, a solution of 30.68 mmol of bim and 50 mL of methanol was added dropwise to the surface. Hereafter, the solvent was allowed to evaporate at 200 °C for 20 min and sonicated in methanol for 30 s at room temperature. The process for ligand solution impregnation was repeated six more times. The repeated ligand impregnation promotes surface functionalization, facilitating nucleation sites for MOF growth [32]. The α-alumina support used in this study presents an average pore size in the macroporous range (typically 100–200 nm), which facilitates the penetration of precursor species and promotes heterogeneous nucleation within the support structure.

#### 3.3.2. ZIF-7 Membrane

A mixture of 6.86 mmol of the linker bim, 15.21 mmol of NaCOOH and 5.14 mmol of Zn(NO_3_)_2_·6H_2_O were dissolved in 40 mL of solvent (DMF or DMF:MeOH). Then, the solution and the modified support were sealed in an autoclave at 120 °C for 3 h. After cooling down to room temperature, the membrane was rinsed with methanol and dried in ambient conditions overnight. Membrane activation was carried out by solvent exchange using methanol. After synthesis, the membranes were immersed in fresh methanol at room temperature for 24 h, replacing the solvent every 8 h, followed by drying at 60 °C overnight, before permeation measurements. This mild activation procedure was selected to avoid rapid solvent removal, which may induce cracking or structural stress in supported ZIF-7 membranes. All membrane syntheses were performed at least in duplicate under identical conditions, and consistent morphological and structural results were obtained, confirming the reproducibility of the synthesis protocol. In this synthesis system, DMF is primarily used as a solvating medium for both the zinc precursor and benzimidazole, while methanol acts as a polar protic cosolvent that modifies solvation interactions and crystallization kinetics [40,44]. Sodium formate was intentionally employed as the main deprotonating agent. Therefore, any contribution of DMF as a deprotonating species is considered secondary under the present conditions, although it has been reported that DMF may generate basic species under solvothermal conditions [58].

### 3.4. Characterization

X-ray diffraction patterns were recorded on a Rigaku Ultima IV Powder X-ray Diffractometer (Rigaku, Tokyo, Japan) equipped with CuKα radiation over a range of 5–30°, using a step size of 2°/min.

A JSM-6510lV (JEOL, Tokyo, Japan) Scanning Electron Microscope was used to analyze the surface morphology as well as the elemental distribution within the ZIF-7 membranes.

Infrared spectroscopic measurements were carried out in a Bruker TENSOR 27 FT-IR Spectrometer (Bruker, Billerica, MA, USA) to analyze the structure of the ZIF-7 membranes.

Thermal degradation measurements were carried out using a TGA, model Netzsch STA 449 F3 Jupiter (NETZSCH Group, Selb, Germany). All samples were scanned from 30 °C to 900 °C at heating rates of 10 °C/min under an N_2_ atmosphere to evaluate the stability of the materials.

The specific surface area was determined by Nitrogen Physisorption measurements at 77° K using an Autosorb iQ equipment (Anton Paar, Graz, Austria), by outgassing samples at 180 °C for eight hours before N_2_ adsorption.

The single-gas permeation setup through ZIF-7 membranes were implemented using the *time-lag* method at feed flow rates of 30 mL/min (Figure S1, Supporting Information).

## 4. Conclusions

Different DMF:MeOH solvent ratios were successfully applied in the synthesis of ZIF-7 crystals and membranes. X-ray diffraction confirmed the formation of the ZIF-7 phase with sodalite topology at a DMF:MeOH ratio of 1:3, whereas higher methanol dilution ratios (1:7 and 1:15) did not yield crystalline structures, likely due to polarity-induced effects that hinder coordination and nucleation processes. Significant differences in crystal size and membrane morphology were observed as a function of solvent composition. The presence of methanol in the synthesis medium modified nucleation and growth kinetics through solvation effects on the benzimidazole linker, resulting in smaller crystals, thinner membranes, and modified intercrystalline packing. These structural features are associated with solvent-mediated heterogeneous intergrowth, including variations in intercrystalline packing, membrane densification, and transport pathway regulation. Although N_2_ adsorption at 77 K resulted in low BET surface areas, these values were interpreted considering the known limitations of nitrogen physisorption for flexible ultramicroporous frameworks. Therefore, the conclusions of this work focus on solvent-mediated membrane growth, morphology evolution, and microstructure-associated transport trends rather than on absolute porosity determination. Accordingly, membrane functionality in the present system is discussed primarily in terms of solvent-mediated microstructural organization and heterogeneous intergrowth behavior. The membrane synthesized with the DMF:MeOH ratio of 1:3 exhibited more differentiated H_2_/CO_2_ transport behavior associated with solvent-mediated intercrystalline organization, showing qualitative agreement with previously reported microstructure-associated transport behavior in ZIF-7 membranes synthesized under DMF-based conditions. Overall, these findings support solvent polarity modulation as an effective physicochemical strategy for regulating heterogeneous nucleation, crystal growth kinetics, membrane densification, intercrystalline organization, and transport pathway evolution during ZIF-7 membrane formation. The present study supports the fundamental role of solvent-mediated coordination, precursor solvation, heterogeneous crystallization behavior, and intercrystalline organization in regulating membrane microstructural evolution and transport pathway organization under reduced DMF conditions. Importantly, these solvent-mediated microstructural effects were achieved while reducing DMF consumption by approximately 75%, highlighting solvent polarity engineering as a promising approach for controlling supported ZIF-7 membrane growth and heterogeneous intergrowth behavior while simultaneously reducing the environmental impact associated with DMF-intensive synthesis routes. Future studies involving operando crystallization analysis, advanced adsorption characterization, and in situ membrane growth monitoring may provide additional insight into the relationship between solvent-mediated microstructural evolution, framework accessibility, and transport pathway organization in supported ZIF-7 membrane systems. Accordingly, the present study is intended primarily as a mechanistic physicochemical analysis of solvent-mediated membrane formation and microstructural evolution under reduced DMF conditions.

## Figures and Tables

**Table 1:** Physicochemical properties of ZIF-7 crystals synthesized with different DMF:MeOH solvent ratios.

Sample	Solvent Ratio	Surface Area (m^2^/g)	Stability (°C)
**ZIF-7-D**	1:0	2.3	450
**ZIF-7-M**	1:3	2.1	500

**Table 2:** Permeance properties of the ZIF-7 membranes, Knudsen constant and kinetic diameter.

Gas	Kinetic Diameter (Å)	Knudsen Constant [Mi/M_H2_]^1/2^	Permeance (mol m^−2^ s^−1^ Pa^−1^) Membrane 1	Qualitative H_2_/Gas Transport Trends Membrane 1	Permeance (mol m^−2^ s^−1^ Pa^−1^) Membrane 2	Qualitative H_2_/Gas Transport Trends Membrane 2
H_2_	2.89	1.00	3.61 × 10^−6^ ${\pm 0.18\times 10}^{-6}$	1.00	4.63 × 10^−7^ ${\pm 0.23\times 10}^{-7}$	1.00
CO_2_	3.30	4.69	4.54 × 10^−7^ ${\pm 0.45\times 10}^{-7}$	7.95	2.89 × 10^−8^ ${\pm 0.29\times 10}^{-8}$	16.02
N_2_	3.64	3.74	6.28 × 10^−7^ ${\pm 0.63\times 10}^{-7}$	5.75	3.98 × 10^−8^ ${\pm 0.40\times 10}^{-8}$	11.63
C_3_H_8_	4.30	4.69	4.98 × 10^−7^ ${\pm 0.50\times 10}^{-7}$	7.24	3.81 × 10^−8^ ${\pm 0.38\times 10}^{-8}$	12.15
C_3_H_6_	4.00	4.58	7.48 × 10^−7^ ${\pm 0.75\times 10}^{-7}$	4.82	7.82 × 10^−8^ ${\pm 0.78\times 10}^{-8}$	5.92

**Table 3:** Comparison of Qualitative H_2_/CO_2_ transport differentiation trends reported for ZIF-7 membranes reported in the literature.

Reference	Method	Solvent	Qualitative H_2_/CO_2_ Transport Differentiation Trends
Li et al., 2010 [31]	in situ growth	DMF	~15
Noh et al., 2015 [33]	in situ growth	DMF	~15.2
Go et al., 2016 [50]	in situ growth	DMF	~15.9
This work (Membrane 1)	in situ growth	DMF	7.9
This work (Membrane 2)	in situ growth	DMF/MeOH (1:3)	16

## Data Availability

The data presented in this study is available on request from the corresponding author.

## Supplementary Materials

The following supporting information can be downloaded at: https://www.mdpi.com/article/10.3390/molecules31132348/s1, Figure S1. Schematic illustration of the single gas permeation setup; Figure S2. FT-IR spectrum of ZIF-7, (a) only DMF and (b) mixture of DMF:MeOH; Figure S3. TG curve of the ZIF-7 powder (a) only DMF and (b) mixture of DMF:MeOH; Figure S4. Elemental mapping by Energy Dispersive X-ray Spectroscopy of the cross section; (a) DMF and (b) DMF: MeOH; Figure S5. Permeation of ZIF-7 membranes as a function of synthesis time.

## References

[1] Caro J., Kustov L.M. (2016). Chapter 7—Supported Zeolite and MOF Molecular Sieve Membranes: Preparation, Characterization, Application A2-Sels, Bert F. Zeolites and Zeolite-Like Materials.

[2] Gascon J., Kapteijn F. (2010). Metal-Organic Framework Membranes—High Potential, Bright Future?. Angew. Chem. Int. Ed..

[3] Li Y., Yang W. (2015). Molecular sieve membranes: From 3D zeolites to 2D MOFs. Chin. J. Catal..

[4] Askari M., Chung T.-S. (2013). Natural gas purification and olefin/paraffin separation using thermal cross-linkable co-polyimide/ZIF-8 mixed matrix membranes. J. Membr. Sci..

[5] Baker R.W. (2002). Future Directions of Membrane Gas Separation Technology. Ind. Eng. Chem. Res..

[6] Dong X., Huang K., Liu S., Ren R., Jin W., Lin Y.S. (2012). Synthesis of zeolitic imidazolate framework-78 molecular-sieve membrane: Defect formation and elimination. J. Mater. Chem..

[7] Kim J., Lee D. (2016). Marked inducing effects of metal oxide supports on the hydrothermal stability of zeolitic imidazolate framework (ZIF) membranes. J. Mater. Chem. A.

[8] Ockwig N.W., Nenoff T.M. (2007). Membranes for Hydrogen Separation. Chem. Rev..

[9] Bux H., Liang F., Li Y., Cravillon J., Wiebcke M., Caro J. (2009). Zeolitic Imidazolate Framework Membrane with Molecular Sieving Properties by Microwave-Assisted Solvothermal Synthesis. J. Am. Chem. Soc..

[10] Park K.S., Ni Z., Côté A.P., Choi J.Y., Huang R., Uribe-Romo F.J., Chae H.K., O’Keeffe M., Yaghi O.M. (2006). Exceptional chemical and thermal stability of zeolitic imidazolate frameworks. Proc. Natl. Acad. Sci. USA.

[11] Banerjee R., Furukawa H., Britt D., Knobler C., O’Keeffe M., Yaghi O.M. (2009). Control of Pore Size and Functionality in Isoreticular Zeolitic Imidazolate Frameworks and their Carbon Dioxide Selective Capture Properties. J. Am. Chem. Soc..

[12] Wu R., Qian X., Rui X., Liu H., Yadian B., Zhou K., Wei J., Yan Q., Feng X.-Q., Long Y. (2014). Zeolitic Imidazolate Framework 67-Derived High Symmetric Porous Co_3_O_4_ Hollow Dodecahedra with Highly Enhanced Lithium Storage Capability. Small.

[13] Cai W., Lee T., Lee M., Cho W., Han D.-Y., Choi N., Yip A.C.K., Choi J. (2014). Thermal Structural Transitions and Carbon Dioxide Adsorption Properties of Zeolitic Imidazolate Framework-7 (ZIF-7). J. Am. Chem. Soc..

[14] Chen E.-Y., Liu Y.-C., Sun T.-Y., Wang Q., Liang L.-J. (2013). Effects of substituent groups and central metal ion on hydrogen adsorption in zeolitic imidazolate frameworks. Chem. Eng. Sci..

[15] Zhang K., Zhang L., Jiang J. (2013). Adsorption of C1–C4 Alcohols in Zeolitic Imidazolate Framework-8: Effects of Force Fields, Atomic Charges, and Framework Flexibility. J. Phys. Chem. C.

[16] Liu Y., Hu E., Khan E.A., Lai Z. (2010). Synthesis and characterization of ZIF-69 membranes and separation for CO_2_/CO mixture. J. Membr. Sci..

[17] Pan Y., Li T., Lestari G., Lai Z. (2012). Effective separation of propylene/propane binary mixtures by ZIF-8 membranes. J. Membr. Sci..

[18] Zhang X.-X., Xiao P., Zhan C.-H., Liu B., Zhong R.-Q., Yang L.-Y., Sun C.-Y., Liu H., Pan Y., Chen G.-J. (2015). Separation of Methane/Ethylene Gas Mixtures Using Wet ZIF-8. Ind. Eng. Chem. Res..

[19] Böhme U., Barth B., Paula C., Kuhnt A., Schwieger W., Mundstock A., Caro J., Hartmann M. (2013). Ethene/Ethane and Propene/Propane Separation via the Olefin and Paraffin Selective Metal–Organic Framework Adsorbents CPO-27 and ZIF-8. Langmuir.

[20] Hara N., Yoshimune M., Negishi H., Haraya K., Hara S., Yamaguchi T. (2014). Diffusive separation of propylene/propane with ZIF-8 membranes. J. Membr. Sci..

[21] Kong L., Zhang X., Liu Y., Li S., Liu H., Qiu J., Yeung K.L. (2014). In situ fabrication of high-permeance ZIF-8 tubular membranes in a continuous flow system. Mater. Chem. Phys..

[22] Krokidas P., Castier M., Moncho S., Sredojevic D.N., Brothers E.N., Kwon H.T., Jeong H.-K., Lee J.S., Economou I.G. (2016). ZIF-67 Framework: A Promising New Candidate for Propylene/Propane Separation. Experimental Data and Molecular Simulations. J. Phys. Chem. C.

[23] Kwon H.T., Jeong H.-K. (2015). Improving propylene/propane separation performance of Zeolitic-Imidazolate framework ZIF-8 Membranes. Chem. Eng. Sci..

[24] Hu Z., Deibert B.J., Li J. (2014). Luminescent metal-organic frameworks for chemical sensing and explosive detection. Chem. Soc. Rev..

[25] Li R., Ren X., Ma H., Feng X., Lin Z., Li X., Hu C., Wang B. (2014). Nickel-substituted zeolitic imidazolate frameworks for time-resolved alcohol sensing and photocatalysis under visible light. J. Mater. Chem. A.

[26] Chizallet C., Lazare S., Bazer-Bachi D., Bonnier F., Lecocq V., Soyer E., Quoineaud A.-A., Bats N. (2010). Catalysis of Transesterification by a Nonfunctionalized Metal−Organic Framework: Acido-Basicity at the External Surface of ZIF-8 Probed by FTIR and ab Initio Calculations. J. Am. Chem. Soc..

[27] Bonitatibus P.J., Chakraborty S., Doherty M.D., Siclovan O., Jones W.D., Soloveichik G.L. (2015). Reversible catalytic dehydrogenation of alcohols for energy storage. Proc. Natl. Acad. Sci. USA.

[28] Karagiaridi O., Lalonde M.B., Bury W., Sarjeant A.A., Farha O.K., Hupp J.T. (2012). Opening ZIF-8: A Catalytically Active Zeolitic Imidazolate Framework of Sodalite Topology with Unsubstituted Linkers. J. Am. Chem. Soc..

[29] Huang X., Zhang J., Chen X. (2003). [Zn(bim)_2_] · (H_2_O)1.67: A metal-organic open-framework with sodalite topology. Chin. Sci. Bull..

[30] He M., Yao J., Li L., Wang K., Chen F., Wang H. (2013). Synthesis of Zeolitic Imidazolate Framework-7 in a Water/Ethanol Mixture and Its Ethanol-Induced Reversible Phase Transition. ChemPlusChem.

[31] Li Y., Liang F., Bux H., Yang W., Caro J. (2010). Zeolitic imidazolate framework ZIF-7 based molecular sieve membrane for hydrogen separation. J. Membr. Sci..

[32] McCarthy M.C., Varela-Guerrero V., Barnett G.V., Jeong H.-K. (2010). Synthesis of Zeolitic Imidazolate Framework Films and Membranes with Controlled Microstructures. Langmuir.

[33] Noh S.-J., Yoon S.P., Han J., Park S., Kim J. (2015). Synthesis and Characterization of ZIF-7 Membranes by *In Situ* Method. J. Nanosci. Nanotechnol..

[34] Li F., Bao X.X., Yu X.F. (2014). Preparation and Gas Permeability of ZIF-7 Membranes Prepared via Two-step Crystallization Technique. Korean Chem. Eng. Res..

[35] Aceituno Melgar V.M., Kwon H.T., Kim J. (2014). Direct spraying approach for synthesis of ZIF-7 membranes by electrospray deposition. J. Membr. Sci..

[36] Jordan A., Hall C.G.J., Thorp L.R., Sneddon H.F. (2022). Replacement of Less-Preferred Dipolar Aprotic and Ethereal Solvents in Synthetic Organic Chemistry with More Sustainable Alternatives. Chem. Rev..

[37] Dong X., Lu D., Harris T.A.L., Escobar I.C. (2021). Polymers and Solvents Used in Membrane Fabrication: A Review Focusing on Sustainable Membrane Development. Membranes.

[38] Marčec J., Ristić A., Logar N.Z. (2024). New Insights into ZIF-90 Synthesis. Molecules.

[39] Ebrahimi M., Mansournia M. (2017). Rapid room temperature synthesis of zeolitic imidazolate framework-7 (ZIF-7) microcrystals. Mater. Lett..

[40] Tu M., Wiktor C., Rosler C., Fischer R.A. (2014). Rapid room temperature syntheses of zeolitic-imidazolate framework (ZIF) nanocrystals. Chem. Commun..

[41] Łuczak J., Kroczewska M., Baluk M., Sowik J., Mazierski P., Zaleska-Medynska A. (2023). Morphology control through the synthesis of metal-organic frameworks. Adv. Colloid Interface Sci..

[42] Goesten M.G., Magusin P.C.M.M., Pidko E.A., Mezari B., Hensen E.J.M., Kapteijn F., Gascon J. (2014). Molecular Promoting of Aluminum Metal–Organic Framework Topology MIL-101 by N,N-Dimethylformamide. Inorg. Chem..

[43] Li H.-M., Yang S.-Y., Wang J.-W., Long L.-S., Huang R.-B., Zheng L.-S. (2010). Coordination steric effect of N,N-dimethylformamide, N,N-dimethylacetamide and N-methyl-2-pyrrolidone on the assembly of coordination polymers. Polyhedron.

[44] Bustamante E.L., Fernández J.L., Zamaro J.M. (2014). Influence of the solvent in the synthesis of zeolitic imidazolate framework-8 (ZIF-8) nanocrystals at room temperature. J. Colloid Interface Sci..

[45] Loubalová I., Kopel P. (2023). Coordination Compounds of Cu, Zn, and Ni with Dicarboxylic Acids and N Donor Ligands, and Their Biological Activity: A Review. Molecules.

[46] Carpenter B.P., Talosig A.R., Rose B., Di Palma G., Patterson J.P. (2023). Understanding and controlling the nucleation and growth of metal–organic frameworks. Chem. Soc. Rev..

[47] Yang Q.-F., Cui X.-B., Yu J.-H., Lu J., Yu X.-Y., Zhang X., Xu J.-Q., Hou Q., Wang T.-G. (2008). A series of metal-organic complexes constructed from in situ generated organic amines. CrystEngComm.

[48] Sapnik A.F., Bechis I., Bumstead A.M., Johnson T., Chater P.A., Keen D.A., Jelfs K.E., Bennett T.D. (2022). Multivariate analysis of disorder in metal–organic frameworks. Nat. Commun..

[49] Kida K., Okita M., Fujita K., Tanaka S., Miyake Y. (2013). Formation of high crystalline ZIF-8 in an aqueous solution. CrystEngComm.

[50] Noguera-Díaz A., Villarroel-Rocha J., Ting V.P., Bimbo N., Sapag K., Mays T.J. (2019). Flexible ZIFs: Probing guest-induced flexibility with CO_2_, N_2_ and Ar adsorption. J. Chem. Technol. Biotechnol..

[51] Arami-Niya A., Birkett G., Zhu Z., Rufford T.E. (2017). Gate opening effect of zeolitic imidazolate framework ZIF-7 for adsorption of CH_4_ and CO_2_ from N_2_. J. Mater. Chem. A.

[52] Zhao P., Fang H., Mukhopadhyay S., Li A., Rudić S., McPherson I.J., Tang C.C., Fairen-Jimenez D., Tsang S.C.E., Redfern S.A.T. (2019). Structural dynamics of a metal-organic framework induced by CO_2_ migration in its non-uniform porous structure. Nat. Commun..

[53] Schneemann A., Bon V., Schwedler I., Senkovska I., Kaskel S., Fischer R.A. (2014). Flexible metal-organic frameworks. Chem. Soc. Rev..

[54] Thommes M., Kaneko K., Neimark A.V., Olivier J.P., Rodriguez-Reinoso F., Rouquerol J., Sing K.S.W. (2015). Physisorption of gases, with special reference to the evaluation of surface area and pore size distribution (IUPAC Technical Report). Pure Appl. Chem..

[55] Ramu G., Lee M., Jeong H.-K. (2017). Effects of zinc salts on the microstructure and performance of zeolitic-imidazolate framework ZIF-8 membranes for propylene/propane separation. Microporous Mesoporous Mater..

[56] Cheng Y., Datta S.J., Zhou S., Jia J., Shekhah O., Eddaoudi M. (2022). Advances in metal–organic framework-based membranes. Chem. Soc. Rev..

[57] Go Y., Lee J.H., Shamsudin I.K., Kim J., Othman M.R. (2016). Microporous ZIF-7 membranes prepared by in-situ growth method for hydrogen separation. Int. J. Hydrog. Energy.

[58] Brozek C.K., Michaelis V.K., Ong T.-C., Bellarosa L., López N., Griffin R.G., Dincă M. (2015). Dynamic DMF Binding in MOF-5 Enables the Formation of Metastable Cobalt-Substituted MOF-5 Analogues. ACS Cent. Sci..

