# Supplementary material for: Solvent Polarity Engineering in Low-DMF ZIF-7 Membrane Growth: Crystallization Behavior, Heterogeneous Intergrowth, and Microstructural Evolution
Source: Molecules. 2026 Jul 3;31(13):2348. doi: 10.3390/molecules31132348 (PMC13362548; doi:10.3390/molecules31132348)
Supplement: Supplementary file 1 [file molecules-31-02348-s001.zip › molecules-4298178-supplementary.pdf]

SUPPORTING INFORMATION

Solvent Polarity Engineering in Low-DMF ZIF-7 Membrane Growth: Crystallization Behavior, Heterogeneous Intergrowth, and Microstructural Evolution

Fernando Romero-Romero [1,2,*], Maria Fernanda Ballesteros-Rivas [1,2], Victor Varela-Guerrero [1,2,*], Sergio Armando Serrano-Palafox [2], Vidal Morales-Mercado [2], Murali Venkata Basavanag Unnamatla[1,2] and José Miguel Arriaga-Merced [2]

[1] Facultad de Química, Universidad Autónoma del Estado de México, Paseo Colón y Paseo Tollocan S/N, Toluca 50120, Estado de México, Mexico; mfballesterosr@uaemex.mx (M.F.B.-R.); mvbasavanagu@uaemex.mx (M.V.B.U.)

[2] Centro Conjunto de Investigación en Química Sustentable UAEM-UNAM, Carretera Toluca-Atlacomulco, km 14.5, Toluca 50200, Estado de México, Mexico; sserranop003@alumno.uaemex.mx (S.A.S.-P.); vmoralesm@uaemex.mx (V.M.-M.); jmiguel_arriagam@yahoo.com.mx (J.M.A.-M.)

* Correspondence: fromeror@uaemex.mx (F.R.-R.), vvarelag@uaemex.mx (V.V.-G.)

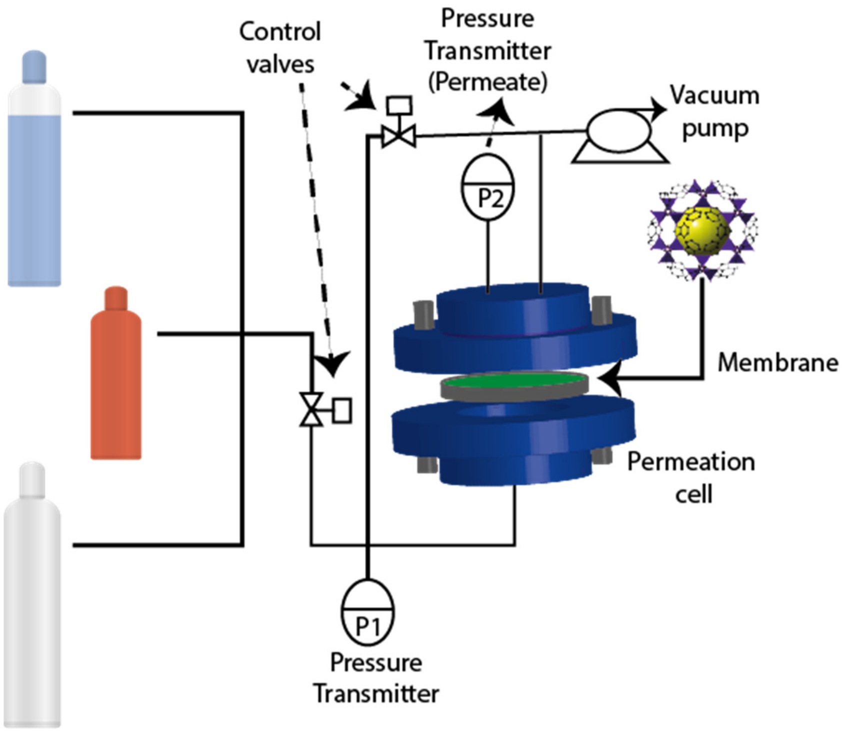

Figure S1. Schematic illustration of the single gas permeation setup.

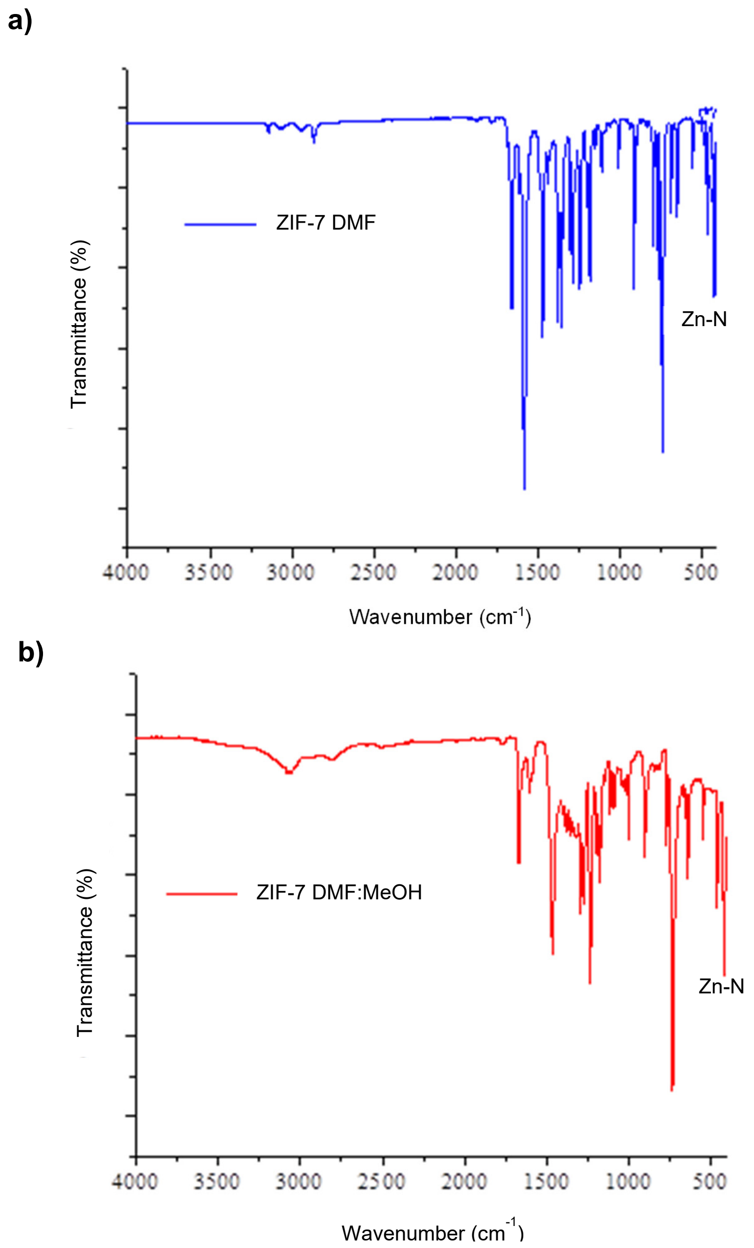

Figure S2. FT-IR spectrum of ZIF-7, a) only DMF and b) mixture of DMF:MeOH.

a)

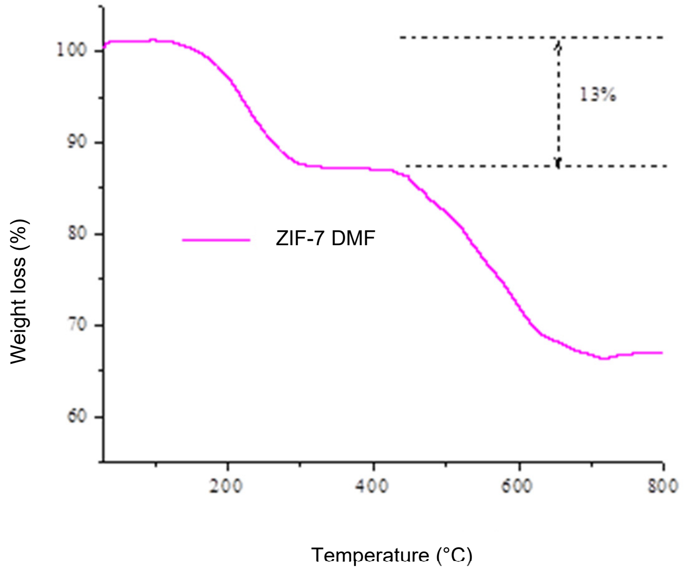

b)

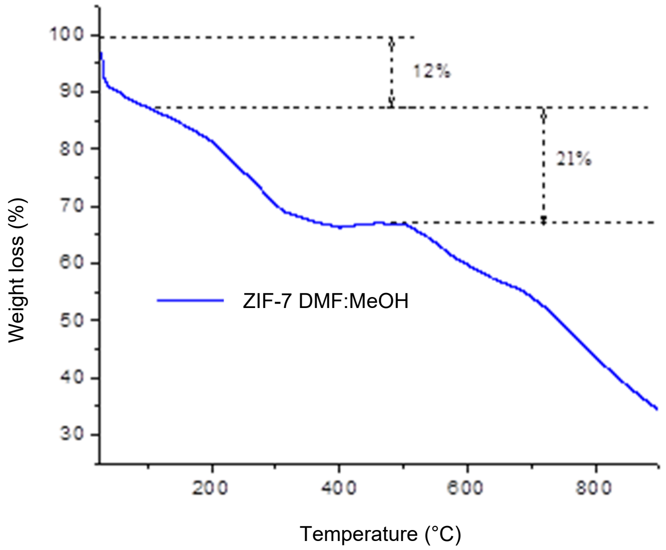

Figure S3. TG curve of the ZIF-7 powder a) only DMF and b) mixture of DMF:MeOH.

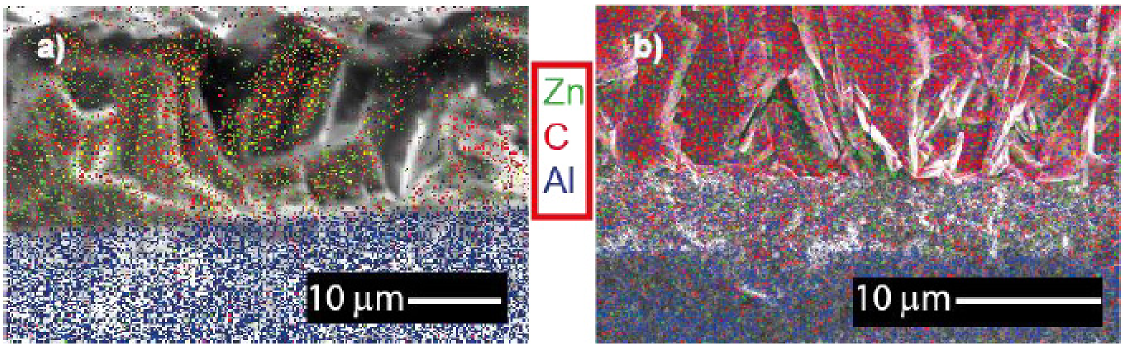

Figure S4. Elemental mapping by Energy Dispersive X-ray Spectroscopy of the cross section; a) DMF and b) DMF: MeOH.

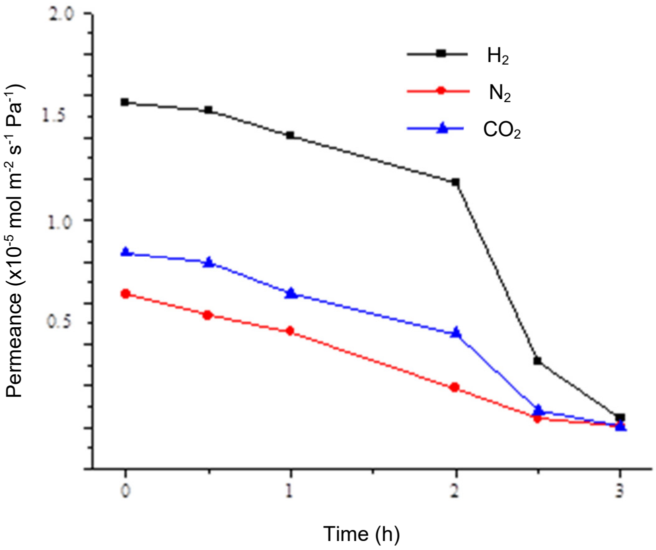

Figure S5. Permeation of ZIF-7 membranes as a function of synthesis time.
